# Editorial: Genetics and Genomics of Polyploid Plants

**DOI:** 10.3389/fpls.2019.00934

**Published:** 2019-07-18

**Authors:** Jun Yang, Zhangying Wang, Yiwei Jiang, Shuizhang Fei

**Affiliations:** ^1^Shanghai Chenshan Plant Science Research Center (CAS), Shanghai, China; ^2^Guangdong Academy of Agricultural Sciences, Guangzhou, China; ^3^Department of Agronomy, Purdue University, West Lafayette, IN, United States; ^4^Department of Horticulture, Iowa State University, Ames, IA, United States

**Keywords:** polyploid, genetics, genomics, evolution, genotype by environment (G × E) interaction

Many of the most economically important crops are polyploids or paleopolyploids. The Research Topic “Genetics and Genomics in Polyploid Plants” focuses on the genome relationships and evolution; inheritance of economically important traits; and development of technologies or methods that facilitate genetics and genomics studies in polyploidy plants. The knowledge gained through studying the evolutionary and population genetics of polyploid crops will advance our understanding of the genetic origin of polyploid crops and its impact on phenotypic variation and facilitate crop improvement. This topic contains 3 reviews, 13 original research articles and 1 method paper.

Kyriakidou et al. reviewed the current genome sequencing strategies in polyploidy plants. Generally, these strategies can also be applied to investigate other organisms with sub-genomes. Ceasar et al. discussed up-to-date genetic improvement, transcriptome analysis and quantitative trait loci mapping using the released genome sequence of finger millet (*Eleusine coracana*). Kandel et al. provided a review update on the available genetic and genomic resources for sugarcane (*Saccharum* spp.) and discussed the challenges in sugarcane biomass improvement. Sharma et al. compared *Saccharum officinarum* and *Saccharum spontaneum* genomes via sequencing of bacterial artificial chromosome libraries. Divergence time estimation suggested that both *Saccharum* spp. experienced independent polyploidization. Dong et al. developed a set of chromosome-specific cytogenetic markers in *Saccharum* spp. via pooled sequencing of bacterial artificial chromosome clones derived from haploid genome of cultivated sugarcane (*S. spontaneum*). Maulana et al. reported quantitative trait loci associated with seedling heat tolerance in winter wheat (*Triticum aestivum*). Daba et al. identified candidate genes that are putatively responsible for determination of kernel weight and kernel length in soft red winter wheat population. Ren et al. studied the genetic architecture of nitrogen-deficiency tolerance in wheat seedlings using a nested association mapping population. Deng et al. examined the chromosome configurations in four artificially constructed pentaploid wheats (BBAAD) and the chromosome instabilities in their progenies. Lu et al. presented us a finely assembled genome of *Arachis ipaensis*, the B-genome progenitor of peanut (*Arachis hypogaea*). As a result, genes related to disease resistance, drought adaptation, nitrogen fixation, and fatty acid biosynthesis were identified and discussed. Clevenger et al. introduced HAPLOSWEEP, the haplotype-based genotyping software, which is applicable in allopolyploid genome analysis and has been applied in *A. hypogaea* as an example. Jia et al. studied accuracies of three Bayes statistical methods on genomic prediction of agronomic traits in alfalfa (*Medicago sativa*). Adhikari et al. suggested that the genetic basis of winter hardiness and fall dormancy in alfalfa were independent and therefore could be improved separately in breeding programs. Wadl et al. investigated the population structure and genetic diversity of the hexaploid sweetpotato (*Ipomoea batatas*) accessions originating from Africa, Australia, Caribbean, Central America, Far East, North America, Pacific Islands, and South America. Taylor et al. performed a genome-wide association study, and significant signals for heading and anthesis were identified in switchgrass (*Panicum virgatum*). At the same time, they also highlighted the potential of manipulating these candidate genes in late-flowering switchgrass breeding. Guo et al. implemented genomic prediction in tetraploid perennial ryegrass (*Lolium perenne*), and gained the highest predictive ability by sequencing depth between 10 and 20 times. Yu et al. presented a hypothetical molecular regulatory network for apetalous trait through the CHR11-PLP pathway in rapeseed (*Brassica napus*).

The research articles featured in this collection covers a range of polyploid plant species including wheat, sugarcane, alfalfa, peanut, sweetpotato, switchgrass, finger millet, perennial ryegrass, and rapeseed. This research collection greatly advanced our understanding of polyploid plants. Many polyploid species are notoriously difficult to study because of their complex genomes, unique reproductive mode, and in some cases long life cycle. Rapid and continued improvements on sequencing capacity and accuracy, computing power, bioinformatics tools, phenotyping throughput and accuracy and other technologies will undoubtedly enhance genetic and genomic research in these species in near future.

## Author Contributions

JY drafted the manuscript. YJ, SF, and ZW edited and revised the manuscript.

### Conflict of Interest Statement

The authors declare that the research was conducted in the absence of any commercial or financial relationships that could be construed as a potential conflict of interest.

